# Relationship Between Patient Activation and Quality of Life in HCN1-Related Epilepsy

**DOI:** 10.14789/ejmj.JMJ25-0064-OA

**Published:** 2026-03-04

**Authors:** MOHIT KUMAR, GEOFFERY IGBERAESE, AHMED ABBAS ZEB, CALUM CONNOLLY, SOHAM KALE, ALI QAYYUM, MAYS AMJAD MUSTAFA HANON, SIDRA HARISS, MOHAMMAD MUSTAFA ABABNEH, TALA ALSYOUTI, RAZAN ZEYAD AL-HAMAIDEH, ZAINA SAMER BASHABSHEH, LINA RABAH ELSAYED

**Affiliations:** 1 Department of Medicine, Rural Medical College, Loni, Maharashtra, India; 1 Department of Medicine, Rural Medical College, Loni, Maharashtra, India; 2 Department of Psychiatry, Middleton St. George Priory, Middleton St. George, UK; 2 Department of Psychiatry, Middleton St. George Priory, Middleton St. George, UK; 3 Department of Medicine, Army Medical College, Rawalpindi, Pakistan; 3 Department of Medicine, Army Medical College, Rawalpindi, Pakistan; 4 Department of Medicine, Colchester General Hospital, East Suffolk and North Essex NHS Trust, Colchester, UK; 4 Department of Medicine, Colchester General Hospital, East Suffolk and North Essex NHS Trust, Colchester, UK; 5 epartment of Medicine, University Hospitals Birmingham NHS Foundation Trust, Birmingham, UK; 5 Department of Medicine, University Hospitals Birmingham NHS Foundation Trust, Birmingham, UK; 6 Department of Stroke Medicine, Royal London Hospital, London, UK; 6 Department of Stroke Medicine, Royal London Hospital, London, UK; 7 Department of Internal Medicine, Prince Hamzah Hospital, Amman, Jordan; 7 Department of Internal Medicine, Prince Hamzah Hospital, Amman, Jordan; 8 Department of General Medicine, Thumbay University Hospital, Ajman, UAE; 8 Department of General Medicine, Thumbay University Hospital, Ajman, UAE; 9 Department of General Medicine, Jordan University of Science and Technology, Irbid, Jordan; 9 Department of General Medicine, Jordan University of Science and Technology, Irbid, Jordan; 10 Department of General Medicine, HMS AlGarhoud Private Hospital, Dubai, UAE; 10 Department of General Medicine, HMS AlGarhoud Private Hospital, Dubai, UAE; 11 Department of General Medicine, Al-Karak Public Hospital, Karak, Jordan; 11 Department of General Medicine, Al-Karak Public Hospital, Karak, Jordan; 12 Department of General Medicine, HMS Mirdif Hospital, Dubai, UAE; 12 Department of General Medicine, HMS Mirdif Hospital, Dubai, UAE

**Keywords:** HCN1-related epilepsy, patient activation, quality of life, seizure frequency, self-management

## Abstract

**Objectives:**

Epilepsies due to variation of the HCN1 gene can be associated with long-term neurological and psychiatric comorbidities, which may impact daily living. The degree of patient empowerment in managing their disease substantially impacts how they assess QoL. This study aimed to investigate the relationship between patient activation and health-related quality of life (HRQOL) in adults with HCN1-related epilepsies.

**Materials and Methods:**

A cross-sectional study was conducted among 403 adult patients with controlled epilepsy attending neurology clinics. Informed consent was taken verbally. Participants completed the Patient Activation Measure (PAM) and Quality of Life in Epilepsy Inventory (QOLIE-31). Descriptive, correlation, and regression analyses were performed using IBM SPSS Statistics version 26.

**Results:**

There was a moderate, positive correlation between patient activation and HRQOL (r = 0.412, p < 0.001). PAM scores were higher in females (p < 0.001), and HRQOL is reported to be better by males (p = 0.010). PAM and QOLIE scores differed significantly with age (p < 0.001), with the highest proportions in the 35-44 and ≥55 years age groups, respectively. Regression analysis showed that greater patient activation (B value 1.85, p < 0.001) and older age (B value 0.48, p = 0.017) were positive predictors of HRQOL. At the same time, female gender, later onset of epilepsy and frequency of seizures were negative predictors.

**Conclusion:**

Increased patient activation is strongly associated with better HRQOL in HCN1-associated epilepsy. Interventions that enhance patient involvement, self-management, and disease awareness may improve psychosocial and clinical outcomes in these patients.

## Introduction

Epilepsy is a long-term neurological disorder marked by repeated, unprovoked seizure episodes that can be seen in individuals of any age worldwide^1)^. Affecting over 70 million people globally, epilepsy occurs bimodally in frequency with peaks during infancy and old age^2)^.

Recent molecular genetic advances have led to the discovery of numerous ion channel mutations in epilepsy syndromes, including HCN1 (hyperpolarisation-activated cyclic nucleotide-gated channel 1), which is emerging as a mutation of increasing prevalence^3)^. HCN channels control rhythmic events in the heart and brain, and recent reports have provided new insights into their ion permeation and voltage gating. Mutations in the HCN1 gene, a significant constituent of heart and brain channel couplets, are responsible for several epileptic syndromes ranging from severe developmental encephalopathies to milder generalised forms^4, 5)^.

Patient activation, defined as an individual's ability and willingness to take care of their own health, plays a significant role in successful self-management and better outcomes with chronic disease^6, 7)^. Increased patient activation is associated with better quality of life (QOL) and lower symptom burden across a variety of chronic conditions, and improving activation levels may also benefit patients^8)^.

Among individuals with epilepsy, greater patient activation was related to more positive partner communication and less severe seizures^9)^. In addition, emotional distress and seizure worry have been reported to be important QoL predictors in epilepsy, and psychosocial factors such as loneliness, coping and stigma may have a bigger predicting power for QoL than clinical variables, emphasising the role of psychological well-being on epilepsy care^10, 11)^.

Although our knowledge of the genetics of HCN1- related epilepsy has advanced, there is little focus on the psychosocial elements that impact patient well-being. Based on the diverse consequences, in addition to a higher mortality rate, patients with HCN1 mutation remain with debilitating epilepsy and cognitive impairment, as well as reduced quality of life. However, the extent to which patients proactively manage their own health - known as patient activation - might be crucial in determining these results. This association has special relevance in HCN1-related epilepsy, for which possible treatments are limited and long-term care is heavily dependent upon cooperation from the patient. By studying the association between patient activation and QOL, this study seeks to emphasise and explain the importance of empowering patients as equals in their care and to provide evidence for incorporating these activated patient principles into comprehensive epilepsy programs.

### Study aims

This research seeks to explore the association between patient activation and HRQOL among people with HCN1-related epilepsy and to determine whether high levels of patient activation are associated with better QoL outcomes in this population.

## Materials and Methods

We performed a cross-sectional observational study to examine the connection between patient activation and QoL in patients with HCN1-related epilepsy. Patients were recruited from the neurology outpatient clinics, representing both public- and private-sector patients, ensuring a diverse patient sample. Data were collected at a single point in time using a structured questionnaire that covered sociodemographic and clinical patient data, as well as items on patient activation and quality of life.

Research assistants were trained to approach the clinically stable HCN1-related epilepsy patients, telling them in simple, non-technical terms about the study and what would be done. All participants provided verbal informed consent before data collection. This method maximised the ability to assess how levels of patient activation are associated with variations in quality of life among people with HCN1-related epilepsy.

### Sampling and eligibility criteria

The population was considered infinite for sample size estimation. Estimating a 50% prevalence proportion (p = 0.5) at a confidence level of 95% (Z = 1.96) and a precision of the estimate or margin of error at approximately 5% (d = 0.05), the sample size required was 384 participants^12)^. Individuals were recruited by convenience sampling through neurology outpatient departments.

Eligible participants were aged 18 years or older, with a confirmed clinical and genetic diagnosis of HCN1-related epilepsy, clinically stable, and able to provide written/verbal consent. Patients with other neurological or psychiatric comorbidities, severe cognitive impairment, communication difficulties, and those who were in status epilepticus needing emergency treatment were excluded. Of the 430 individuals interviewed, 400 respondents consented, qualified, and completed the structured questionnaire. The use of convenience sampling facilitated rapid recruitment in specialised clinical settings, at the potential expense of selection bias and generalizability to the broader population of people with epilepsy.

### Instruments and measures

A specifically designed structured questionnaire was utilised to gather detailed information for this study. It consisted of 3 principal sections: (1) demographic and clinical characteristics, (2) the Patient Activation Measure (PAM), and (3) the Quality of Life in Epilepsy Inventory (QOLIE-31). All instruments were used in their original English translations, with no language or cultural adaptations.

### Demographic information

The initial section collected participants' demographics and clinical information, i.e. subgroup and correlation with QoL outcomes. The age, sex, marital status, educational level, occupation, duration of seizure disorder, medication history and whether an immediate family member had a neurological disease were included. These variables were included to describe the study population and to explore potential correlates of patient activation and QoL in people with HCN1-related epilepsy.

### Patient activation measure (PAM)

The PAM, developed by Hibbard et al. (2004), was applied to assess individuals' knowledge, skills, and confidence in managing their own health. The PAM is a 13-item scale that evaluates patients' engagement across four levels of activation: passive recipients of care, passive, active, and self-managers. Each of them will be rated on a 4-point Likert scale (strongly disagree, strongly agree) to produce an overall score, which will be converted to a 0-100 activation score. Higher scores will reflect greater patient activation. The PAM has been widely tested in chronic disease groups and is highly internally consistent (Cronbach's α > 0.85). The presence of self-management ability among adults with HCN1- related epilepsy in this study was possible only through its inclusion in this study^13)^.

### Quality of life in epilepsy inventory (QOLIE-31)

The QOLIE-31, a disease-specific measure developed by Cramer et al. (1998), was used to measure health-related quality of life. There is a 31-item instrument with seven domains: worry about seizures, emotional well-being, energy/fatigue, cognitive functioning, medication effects, social functioning, and overall QOL. All items are rated on a Likert-type scale, and the raw scores will be converted to a 0-100 scale, with higher scores indicating a higher quality of life. QOLIE-31 has been extensively tested in populations with epilepsy and has demonstrated high internal reliability (Cronbach's alpha 0.77 to 0.90). It was used in this study to provide a thorough assessment of the relationships among patient activation, psychosocial health, and functional health in individuals with HCN1-related epilepsy^14)^.

### Data collection procedure

Eligible participants were recruited during their routine visits to the neurology outpatient departments. A structured questionnaire comprising demographic data, PAM, and QOLIE-31 was used to collect data over six months, from March to August 2025. Respondents were asked to complete the questionnaire on their own or with guidance from trained investigators, depending on their literacy and preference. Every answer was thoroughly checked for completeness and accuracy. This culturally sensitive process was standardised, ensuring high-quality data collection and participant comfort throughout the study.

### Analytical procedures

The data were processed and analysed using IBM SPSS Statistics, version 26 (IBM Corp.; Armonk, NY, USA). Research on demographic and clinical characteristics of participants was summarised using descriptive statistics, such as frequencies and percentages. The Kolmogorov-Smirnov and Shapiro-Wilk tests were used to assess the normality of continuous variables, such as the total scores of the PAM and QOLIE. Since both variables were normally distributed, parametric tests were used. The Pearson correlation coefficient was used to test the association between PAM and QOLIE scores. Independent-samples t-tests were used to compare gender differences in mean scores, whereas age- related differences were analysed using a one-way ANOVA. The t-tests and ANOVA results reported effect sizes as Cohen's d and partial eta-squared (η^2^), respectively. A multiple linear regression analysis followed this to identify predictors of QOLIE scores, including patient activation, age, gender, age at epilepsy onset, seizure frequency, and type of HCN1 mutation. A p-value below 0.05 was regarded as statistically significant.

### Ethics & compliance

The study protocol was already approved by the Institutional Review Board of Royal London Hospital, London, UK (IRB reference no. RLH-RSRCH/IRB- 25-0746) and met the ethical requirements governing the conduct of a research study and the protection of the information stored against disclosure. The privacy, dignity, and confidentiality of all participants were preserved during the research process. All the data obtained were coded and kept in a secure location, with access restricted to the principal investigator and selected members of the research team. The subjects were advised of their right to withdraw from the study at any time without affecting their further medical care. These ethics ensured the research was conducted transparently and with full respect for the rights and well-being of the participants.

## Results

### Baseline demographic and clinical characteristics of participants (N = 403)

Table 1 summarises the demographic data for 403 respondents. The majority were aged 35-44 years (N = 142, 35%) and 45-54 years (N = 113, 28%), with slightly more males (N = 210, 52%) than females (N = 193, 48%). Participants who were married accounted for 32% (N = 131), and those who were single, divorced/separated, or widowed accounted for 24% (N = 97), 27% (N = 107), and 17% (N = 68), respectively. Primary education had been the most prevalent (N = 160, 40%), and most were unemployed (N = 142, 35%) or homemakers (N = 99, 25%). The onset of epilepsy was mainly in the 6-10 years (N = 128, 32%) and 11-15 years (N = 146, 36%) range. Frequencies of seizures 4-10 times over six months were usual (N = 142, 35%). 44% (N = 176) took two anti-seizure drugs, and the most common type of HCN1 mutation was that of uncertain significance (N = 170, 42%). 50% of respondents reported no family history of epilepsy (N = 200, 50%).

**Table 1:** Baseline demographic details of the sample (N = 403)

Variable	f (N)	%		Variable	f (N)	%
Age Group				1-5 years	115	28
18-24 years	42	10		6-10 years	128	32
25-34 years	68	17		11-15 years	69	17
35-44 years	142	35		> 15 years	29	7
45-54 years	113	28		Duration of epilepsy		
55 years or above	38	9		< 5 years	58	14
Gender				5-10 years	121	30
Male	210	52		11-15 years	146	36
Female	193	48		> 15 years	78	19
Marital status				Seizure frequency (past 6 months)		
Single	97	24		None (seizure-free)	61	15
Married	131	32		1-3 times	136	34
Divorced/ separated	107	27		4-10 times	142	35
Widowed	68	17		More than 10 times	64	16
Educational level				Current anti-seizure medications		
No formal education	70	17		None	37	9
Primary	160	40		1 medication	112	28
Secondary	121	30		2 medication	176	44
Higher secondary	41	10		3 or more medications	78	19
Bachelor's degree or above	11	3		Type of HCN1 mutation (if known)		
Employment status				Pathogenic	30	7
Student	29	7		Likely pathogenic	118	29
Employed	91	23		Variant of uncertain significance	170	42
Home-maker	99	25		Not tested/unknown	85	21
Unemployed	142	35		Family history of epilepsy		
Retired	42	10		Yes	66	16
Age at epilepsy onset				No	200	50
< 1 year	62	15		May be	137	34

Note. f = frequency, % = percentage; Values are presented as N (%), N = 403; No statistical comparisons were performed for demographic variables in this table

### Correlation between patient activation and quality of life scores (N = 403)

Table 2 presents the Pearson correlation coefficients for the total PAM and QOLIE scores of 403 participants. The correlation was significant (r = 0.412, t(401) = 9.06, p < 0.001), and quality of life was higher at higher levels of patient activation.

**Table 2:** Pearson correlation between total patient activation and QOL scores (N = 403)

Variables	Total patient activation measure questionnaire (PAM)	Total quality of life in epilepsy (QOLIE)
Total patient activation measure questionnaire (PAM)	-	-
Total quality of life in epilepsy (QOLIE)	r = 0.412, t(401) = 9.06, p < 0.001**	-

Note. Values represent Pearson correlation coefficients (r) between continuous variables; N = 403; **= p < 0.001 was considered statistically significant.

### Gender differences in patient activation and quality of life among individuals with epilepsy (N = 403)

Table 3 shows the gender differences in both the PAM and QOLIE scores for 403 participants. The PAM scores were markedly higher among females (M = 27.32, SD = 3.15) than among males (M = 25.84, SD = 3.40), t (401) = -3.72, p < 0.001, with a medium effect size (Cohen's d = 0.45). On the other hand, males (M = 97.85, SD = 7.50) reported higher QOLIE scores than females (M = 95.24, SD = 7.10), with a small-to-medium effect size (Cohen d = 0.36), suggesting that gender may affect patient activation and QOL in epilepsy.

**Table 3:** Gender differences in patient activation and QOL among individuals with epilepsy (N = 403)

Variable	Male (N = 210 ; 52%) M±S.D	Female (N = 193 ; 48%) M±S.D	t	p	Cl 95%		Cohen's D
					LL	UL	
Total patient activation measure questionnaire (PAM)	25.84 ± 3.40	27.32 ± 3.15	-3.72	< 0.001***	-2.26	-0.70	0.45
Total quality of life in epilepsy (QOLIE)	97.85 ± 7.50	95.24 ± 7.10	2.58	0.010**	0.62	4.60	0.36

Note. Values are presented as Mean ± Standard Deviation; Independent samples t-tests were conducted to compare participants of both genders; Group sizes are shown as N (%); Reported statistics include p-values, t-values, 95% Confidence Intervals (CI), and effect sizes (Cohen's d); A p-value < 0.01, < 0.001 was considered statistically significant, N = 403.

### Age-related differences in patient activation and quality of life in epilepsy patients (N = 403)

Table 4 presents age differences in PAM and QOLIE scores for 403 participants. The differences were significant among the age groups for both PAM (F(4,398) = 12.37, p < 0.001, eta = 0.11) and QOLIE (F(4,398) = 10.30, p < 0.001, eta = 0.09). The highest PAM score was observed among participants aged 35-44 years (M = 28.10, SD = 3.00), and QOLIE scores increased with age. Still, the highest score was observed among participants aged 55 and above (M = 26.7, SD = 4.1), indicating that patients become more active and have the highest quality of life at this age.

**Table 4:** ANOVA results showing age-related differences in patient activation and quality of life (N = 403)

Variable	18-24 years (N = 82 ; 10%); M ± S.D	25-34 years (N = 94 ; 17%); M ± S.D	35-44 years (N = 118 ; 35%); M ± S.D	45-54 years (N = 71 ; 28%); M ± S.D	55 years & above (N = 38;9%); M ± S.D	F(4,398)	p	η^2^
Total patient activation measure questionnaire (PAM)	25.40 ± 3.60	26.70 ± 3.25	28.10 ± 3.00	27.60 ± 3.10	25.10 ± 3.45	12.37	< 0.001***	0.11
Total quality of life in epilepsy (QOLIE)	20.6 ± 4.2	21.4 ± 4.5	23.1 ± 4.6	25.2 ± 4.3	26.7 ± 4.1	10.30	< 0.001***	0.09

Note. Data are presented as Mean ± Standard Deviation (M ± SD); Group sizes are shown as N (%). One-way ANOVA was conducted to examine the effect; All comparisons were significant at p < 0.001; η^2^ represents the partial eta-squared effect size.

### Multiple linear regression predicting quality of life (QOLIE) from patient activation and clinical variables (N = 403)

Table 5 presents the results of a multiple linear regression predicting QOLIE scores in 403 patients, using patients' activation and clinical variables. The Total Patient Activation Measure (PAM) scores were significant predictors (B = 1.85, 0.38 = -0.001), indicating that higher activation levels are associated with higher QOL. QOLIE was also positively related to age (B = 0.48, 0.13, p = 0.017). On the other hand, female gender (B = -2.12, -0.10, p = 0.011), later age at epilepsy onset (B = -0.68, -0.15, p = 0.007), and higher seizure frequency within the past six months (B = -1.45, -0.22, p < 0.001) were significant negative predictors. These results indicate that patient-related, as well as clinical, factors have an independent effect on the QOL of epilepsy patients.

**Table 5:** Multiple linear regression predicting quality of life (QOLIE) from patient activation and clinical variables (N = 403)

Model	B	SE	β	t	p	95% CL	
						LL	UL
Constant (total quality of life in epilepsy)	42.315	5.842	-	7.24	<0.001***	-	-
Total patient activation measure (PAM)	1.85	0.28	0.38	6.61	<0.001***	1.30	2.40
Age (years)	0.48	0.20	0.13	2.40	0.017***	0.09	0.87
Gender (Male = 1, Female = 2)	-2.12	0.83	-0.10	-2.55	0.011**	-3.75	-0.49
Age at epilepsy onset	-0.68	0.25	-0.15	-2.72	0.007**	-1.18	-0.19
Seizure frequency (past 6 months)	-1.45	0.33	-0.22	-4.39	<0.001***	-2.10	-0.80

Note. Multiple linear regression was conducted to identify predictors. Scores; Values include unstandardized coefficients (B), 95% confidence intervals (CI), standard error (SE), standardised beta coefficients (β), and p-values; **p < 0.01, *** p < 0.001 were considered statistically significant, N = 403.

### Distribution of simulated total QOLIE scores reflecting regression model coefficients (N = 403)

**Figure 1:**
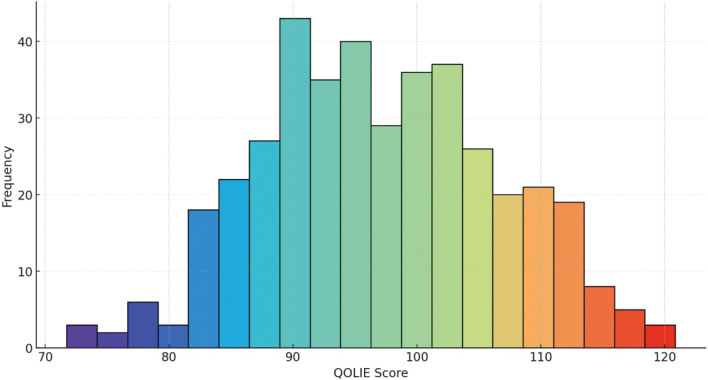
Histogram of stimulated total QOLIE produced to reflect regression model coefficients (N = 403)

Figure 1 shows a histogram of simulated total QOLIE scores for 403 individuals, along with the regression coefficients. The distribution is approximately normal but slightly right-skewed, with scores between 85 and 105. The highest frequency of respondents is approximately 95-90, indicating that a significant number responded at moderate levels of quality of life. There were fewer participants at the very low (under 80) and very high (above 115) ends, indicating limited representation of the very poor and the very high-quality-of-life experiences. In general, the histogram shows that the majority of patients with epilepsy in this model have mid-range QOLIE scores, with a gradually decreasing frequency of scores above the mean.

## Discussion

The current paper has investigated the association between patient activation and health-related quality of life (HRQOL) among participants with HCN1-related epilepsy. We found a moderate positive relationship between total patient activation and QOL (r = 0.412, p < 0.001), such that the more activated patients were in managing their epilepsy, the better they reported their QOL. These results are consistent with previous findings, in which patients who experienced greater reductions in seizures displayed QOLIE scores reflecting the association between active disease intervention and enhanced quality of life^15)^.

Our results show that female respondents reported significantly higher Patient Activation levels than male respondents (Cohen's d = 0.45), suggesting greater active engagement in their condition management. Consistent with earlier research showing that women were more likely than men to report high patient activation, our findings also revealed similar gender differences across a diverse sample of health engagements^16)^. Males in our sample reported a slightly better QOL compared to females (t = 2.58, p = 0.010; Cohen's d: 0.36). Conversely, a previous publication found no substantial gender differences in QOL in epilepsy, thus reflecting that gender effects on QOL may differ depending on the subtypes of epilepsy under study and populations^17)^.

Our investigation found that age had a significant effect on patient activation, with the largest PAM scores reported in those aged 35-44 years (F = 12.37, p < 0.001, η^2^ = 0.11). While earlier studies did not stratify by age, this also suggests that higher patient activation levels are associated with greater benefits in HC and QoL, indicating the value of PAM in assessing patient engagement^18)^. In our study, quality of life increased with age, and the best responders were within the age range of participants (55 years or older). The findings are in line with studies demonstrating that older participants report higher levels of life satisfaction and positively valenced subjective well-being than younger counterparts^19)^.

Better patient activation (PAM) was associated with higher QOLIE scores, similar to a previous study showing that patients who experienced a larger reduction in seizures - indicative of greater involvement - reported higher QOLIE scores^14)^. Female sex was a negative predictor of QOLIE scores. However, the effect was small, which is supported by previous literature that has found gender effects on quality of life may be epilepsy subtype- and population-specific^17)^. Older age was associated with slightly higher QOLIE scores, consistent with the argument that subjective well-being may increase with age^19)^. In our study, later age at epilepsy onset was significantly associated with poorer QoL in late-onset patients, consistent with previous studies showing that older patients are affected by many functional, social, and comorbidity-related issues that impact QoL^20)^. In our research, high seizure frequency over the last six months was associated with poorer QOL, a statistically significant association. This is consistent with an earlier investigation, which demonstrated that the patients who experienced seizures more often expressed poorer QoL and other correlates, such as depression, stigma and medication-related undesirable effects^21)^.

Taken together, these data underscore the importance of patient activation, seizure control, age and age at onset as significant predictors of QOL in HCN1-related epilepsy with implications for interventions for the active management of disease and reduction of seizures to enhance patient well-being.

### Study constraints

A few limitations must be noted. The cross-sectional design precludes drawing causal conclusions about the link between patient activation and QOL. Longitudinal research is necessary to determine whether intensifying activation results in lasting improvements in well-being. Second, selecting the sample through convenience sampling from neurology OPDs in two cities might limit generalizability to rural populations or patients with severe disease profiles. Third, all the information was gathered via self-report instruments, and recall and social desirability biases cannot be ignored. Furthermore, the English version of the questionnaires was used, and, as it lacked local validation/local translation, cultural or linguistic differences could also have influenced responses. Third, clinical variables, including seizure type, medication adherence, and cognitive status, were not independently validated from clinical records, which might have reduced precision.

### Future recommendations

Further studies employing longitudinal or intervention designs are warranted to determine whether improved patient activation from educational or digital self-management interventions is associated with changes in seizure control and quality of life. Cross-validation of patient activation and QOL measures in local languages would increase the utility and generalizability of results across different populations. Additionally, qualitative studies examining patients' views might provide a more thorough understanding of the barriers and facilitators to activation in genetic epilepsies. Lastly, incorporating patient activation concepts into precision medicine paradigms for HCN1-related epilepsy may enhance personalised care, patient involvement, and outcomes.

### Conclusion

In summary, this study indicates that patient activation is strongly related to HRQL in patients with HCN1-related epilepsy. Psychological involvement and self-management ability are important factors for patients' well-being beyond clinical and genetic factors. Encouraging patient activation through structured education, counselling, and self-efficacy development may thus constitute a valuable approach to comprehensive epilepsy care. Empowering patients to become active participants in their healthcare might improve the QOL and long-term clinical outcomes for this challenging genetic disorder.

## Data availability

The datasets generated and analysed during the current study are available from the corresponding author on reasonable request.

## Author contributions

MK conceived and designed the study and coordinated the overall project. CC contributed to study conceptualisation, manuscript review, and refinement of clinical interpretations. GI contributed to the study design, patient-related assessments, and understanding of clinical findings. AAZ assisted in data collection, literature sourcing, and initial drafting support. SK supported patient recruitment and data collection. AQ performed the statistical analysis, interpreted the results, and served as the corresponding author. MAMH participated in the literature review and drafting of the introduction and background. SH assisted with data entry and data quality assurance. MMA contributed to methodology development and manuscript revision. TA assisted with data management and organisation of study variables. RZAH contributed to the formatting and preparation of tables and figures. ZSB participated in proofreading and critical revision of the manuscript. LRE contributed to the literature review, manuscript editing, and final approval of the draft.

## Conflicts of interest statement

The authors declare that there are no conflicts of interest.
